# The Asthma-COPD Overlap Syndrome: A Common Clinical Problem in the Elderly

**DOI:** 10.1155/2011/861926

**Published:** 2011-10-30

**Authors:** Amir A. Zeki, Michael Schivo, Andrew Chan, Timothy E. Albertson, Samuel Louie

**Affiliations:** ^1^Division of Pulmonary, Critical Care and Sleep Medicine, Department of Internal Medicine, University of California, Davis, CA 95616, USA; ^2^Genome and Biomedical Sciences Facility (GBSF), Center for Comparative Respiratory Biology and Medicine (CCRBM), Davis, CA 95616, USA; ^3^VA Northern California Health Care System, Martinez, CA 94553, USA

## Abstract

Many patients with breathlessness and chronic obstructive lung disease are diagnosed with either asthma, COPD, or—frequently—mixed disease. More commonly, patients with uncharacterized breathlessness are treated with therapies that target asthma and COPD rather than one of these diseases. This common practice represents the difficulty in distinguishing these disorders clinically, particularly in patients with a history that does not easily differentiate asthma from COPD. A common clinical scenario is an older former smoker with partially reversible or fixed airflow obstruction and evidence of atopy, demonstrating “overlap” features of asthma and COPD. We stress that asthma-COPD overlap syndrome becomes more prevalent with advancing age as patients respond less favorably to guideline-recommended drug therapy. We review the similarities and differences in clinical characteristics between these disorders, and their physiologic and inflammatory profiles within the context of the aging patient. We underscore the difficulties in differentiating asthma from COPD in current or former smokers, share our institutional experience with overlap syndrome, and highlight the need for new research to better characterize and investigate this important clinical phenotype.

## 1. Introduction

The general internist, allergist, and pulmonologist commonly encounter adult patients with clinical features of both asthma and chronic obstructive pulmonary disease (COPD). Differentiating the underlying cause of their symptoms becomes difficult and often leads to blanket therapy directed towards airway hyperreactivity (AHR), airway inflammation, airflow obstruction, and allergic disease. A salient example is an older patient with a history of seasonal allergies and asthma, a current or past smoking history, and progressive symptoms of acute-on-chronic dyspnea. They may demonstrate fixed airflow obstruction or partial reversibility on spirometric testing, an elevated total IgE, and a slightly increased nitric oxide level. Does such a patient have COPD with AHR, remodeled asthma that has progressed to partially reversible or “fixed” airflow obstruction, or overlapping COPD and asthma—the so-called *asthma-COPD overlap syndrome*? Is some degree of airflow obstruction simply related to natural aging of the lung (a decline in FEV_1_ exceeding the predicted 25 to 30 mL per year), and should this be treated? These questions beget further questions regarding the need to distinguish the components of asthma-COPD overlap and the associated treatment implications, including drug side effects, compliance, and variable response to drug therapy with advancing age. This becomes even more relevant in the elderly population where the overlap syndrome is prevalent.

In this paper we review the data which address the biologic, clinical, and diagnostic differences between asthma and COPD. We will also highlight several areas of overlap between these disorders supporting the need to clinically define this subgroup of patients with obstructive lung disease. Further, we aim to define the overlap syndrome based on the current understanding in the literature and our clinical experience and practice, while underscoring the challenges that remain in understanding this important entity. Ultimately, we propose a work-up and treatment algorithm which fits our patient population, and we raise important questions for future studies needed to advance the recognition and management of asthma-COPD overlap.

## 2. Asthma versus COPD

### 2.1. Accepted Differences

In theory, asthma and COPD are different diseases each with a unique natural history and pathophysiology. The *British Hypothesis* holds that asthma and COPD are distinct diseases that develop by unique mechanisms [1]. It is widely accepted that asthma generally manifests as *intermittent* and *reversible* airway obstruction, whereas COPD is *progressive* and *irreversible * [2–4]. Based on current guidelines, the postbronchodilator response in asthma shows *complete reversibility* of airway obstruction. In COPD, there is either *no reversibility *(i.e., fixed obstruction) or there is *partial reversibility* of airway obstruction following bronchodilator, described as “COPD with partial reversibility” [5]. In this latter entity, one can demonstrate reversibility as an improvement in lung function, but the patient remains obstructed on spirometric measurements (hence, the designation of “COPD” rather than “asthma”). Preserved carbon monoxide diffusion capacity (DL_CO_) on PFT and a higher ratio of airway-to-lung parenchymal abnormalities (on lung imaging by high-resolution chest tomography) may also distinguish asthma from COPD [6].

Clinically, the distinction between asthma and COPD is most apparent at the extremes of age [7], where younger patients tend to have more asthma symptoms and older patients (age > 60) tend to have COPD symptoms. A history of cigarette smoking and evidence of emphysema in an older patient with spirometric airflow obstruction would favor COPD. A nonsmoking younger patient with a history of childhood asthma or wheezing and atopy with reversible airflow obstruction would favor asthma. Though symptoms can often overlap, Beeh and colleagues developed a questionnaire to differentiate asthma and COPD [8]. On a scale of 1 to 15, the questionnaire performed best at a cutoff of 7 with a sensitivity of 87.6% for COPD, though ~20% of patients had overlap features (scores 6–8). Practically speaking for the clinician, the distinction between asthma and COPD is largely based on clinical findings.

Major differences exist in the structural and inflammatory signatures of asthma and COPD when studied in isolated, well-defined populations [9–12]. These include elevated IgE, induction of T_h_2 cells, eosinophilic infiltration, reticular basement membrane thickening, and smooth muscle hyperplasia in asthma. In contrast, increased neutrophils, induction of T_h_1 and T_h_17 cells, TGF*β*-induced small airway fibrosis, goblet cell hyperplasia, and MMP elastic tissue destruction are typically found in COPD [13, 14]. Bronchoscopic endobronchial biopsies have shown smooth muscle cell hypertrophy and chronic inflammation in asthma [10, 15, 16]. Endobronchial biopsy has also distinguished two inflammatory subtypes of severe asthma [17]. Though there are major histologic differences between asthma and COPD, endobronchial biopsy is not widely used in the workup and management of obstructive lung disease [18] as the risk-benefit profile is often unfavorable. Animal and human studies suggest that cytokine profiles may be used to help distinguish between asthma and COPD. However, these data also indicate that a disease continuum exists in some patients with elements of both diseases [1]. Ongoing research using exhaled nitric oxide and other exhaled biomarkers may provide reliable biological parameters to aid in diagnosis [19–22]. 

Existing treatment options for asthma and COPD highlight some important differences [23, 24]. For example, COPD with reduced DL_CO_ may qualify for supplemental oxygen therapy [25] and referral to a pulmonary rehabilitation program [26, 27]. Severe allergic asthma with an appropriate high level of serum IgE may qualify for treatment with omalizumab [28, 29], and, in those with allergic asthma not well controlled on ICS, leukotriene receptor antagonists [30] or 5-lipoxygenase inhibitors may improve symptoms. Bronchial thermoplasty, a novel therapy that uses endobronchial radiofrequency ablation to reduce airway smooth muscle mass, reduces asthma symptoms and rates of exacerbation [31]. However, the link between clinical classification or diagnosis, pathophysiology, and guideline-based treatments can be opaque in practice due to the lack of simple tests that can reliably distinguish the two diseases. Clinicians will consequently lump together asthma and COPD treating them as one condition. 

### 2.2. Difficult Separation, Supporting an Overlap Syndrome

Many clinicians recognize that asthma and COPD can appear more similar than dissimilar clinically. Typical symptoms of dyspnea, wheezing, or cough do not have the sensitivity or specificity to distinguish these two disorders among older adults after excluding comorbid conditions such as heart failure, diastolic dysfunction, aspiration, GERD, or vocal cord dysfunction. According to Soriano et al., asthma and COPD are difficult to differentiate because (1) the conditions are viewed as part of a disease continuum; (2) they have strong overlapping features; (3) there is no incentive to differentiate whether their treatment and prognosis are the same; (4) there is a lack of clear guidelines as to how the distinction can be made in clinical practice; (5) uncertain criteria are used by physicians to classify patients as having asthma or COPD [7]. Despite years of research and the prolific expansion of guidelines from both European and American respiratory societies, distinguishing these two common diseases remains a daunting challenge. 

Recent reviews underscore the pitfalls in diagnosis, management, and treatment of overlap syndrome, asthma, and COPD [32–35]. The *Dutch Hypothesis* maintains that asthma and AHR predispose patients to develop COPD later in life [36] and that asthma and COPD are different expressions of a single disease (based on the timing of environmental and epigenetic influences amidst a common genetic background). Some authorities argue that obstructive lung disease is a progressive disease that begins in early childhood, where COPD is the final manifestation. Recent epidemiologic findings, from a long-term cohort study in the United States, point to asthma as a significant risk factor for the future development of COPD [37]. Unless there are clear exposures, such as a prolonged smoking history in a person with severe emphysema, clinicians recognize that considerable phenotypic heterogeneity makes clear distinctions between obstructive lung diseases problematic [38, 39]. These challenges are nicely demonstrated by a series of pro-con debates between Kraft and Barnes [40, 41].

Not all comparisons between asthma and COPD pathology show unique structural differences. In 100 select patients with clinically determined asthma and COPD who underwent endobronchial biopsies, there were no statistically significant differences in key pathologic features [18]. Though eosinophilic infiltration and basement membrane thickening were associated with asthma, the overall differences in these features, metaplasia, and epithelial inflammation did not allow for pathologic differentiation. Airway remodeling and the lung's specific repair responses may account for some of the pathological similarities reported in asthma and COPD [42, 43]. These structural similarities in small airways may contribute to the observed clinical overlap. Up to 50% of COPD patients can have AHR due to the narrowing of their distal airways and predisposition to bronchospasm [3]. However, Fabbri et al. showed that despite a similar degree of fixed airway obstruction, asthmatic and COPD airway inflammation and structural changes do not appear the same [12]. But as imaging and airflow resistance assessment techniques improve, the small airway changes associated with asthma and COPD which appear dissimilar may result in a phenotypically similar outcome [44]. Limitations inherent to the different patient cohorts studied and research methodologies used may confound and limit final conclusions regarding a clear histopathologic-physiologic link.

Asthma and COPD can overlap in their inflammatory sputum profiles and lung function. Severe bronchial asthma with fixed obstruction has an increased number of neutrophils similar to COPD [45]. Eosinophilic inflammation in COPD may play a substantial role and be associated with greater postbronchodilator reversibility [46]. Chronic bronchitis or emphysema patients with airway eosinophilia demonstrate increased airway reversibility and respond more readily to corticosteroid therapy [47–49]. In smokers with severe obstructive bronchitis, sputum eosinophilia predicts a beneficial response to prednisone treatment [50]. Evidence of bronchodilator response (i.e., reversibility) or AHR may be an important measure not only for diagnosis but also for prognosis with respect to the rate of lung function decline and asthma mortality [51, 52]. However, not all studies agree that bronchodilator responses correlate with the subsequent rate of lung function decline [53]. Some authors found that the prognosis in terms of all-cause mortality is strongly correlated with age, smoking, and the best attainable FEV_1_ regardless of reversibility [54]. Given that overlap features become prevalent with increasing age and a smoking history, there are important implications for prognosis regarding this syndrome.

According to Diaz-Guzman et al., the treatment of asthma and COPD in older adults does not differ from available guidelines but may be complicated by the presence of comorbidities [32]. However, this position fails to grasp the highly variable response to anti-inflammatory ICS therapy in COPD and in asthma where up to 46% of older patients with moderate disease do not respond at all to beclomethasone [55]. The long-term consequences in nonresponding asthmatics chronically treated with ICS are largely unknown but may predispose this cohort to more frequent acute exacerbations and lung function decline over time. This is complicated in the elderly adult who may have overlap features and does not respond to therapy. 

Asthma-COPD overlap may have origins in childhood, for example, infections, atopy, and tobacco-smoking exposure (Figure 6) but escapes earlier diagnosis because of current definitions and guideline-based approaches to chronic obstructive lung diseases. This could result in a heuristic bias potentially overlooking patients in their 40s and 50s with overlap syndrome by labeling them as lone “COPD” or partially reversible “asthma.” This becomes even more relevant in the elderly population (age ≥ 65 years) where the overlap syndrome is increasingly recognized because of variable response to guideline drug therapy, more frequent healthcare resource utilization [32–35, 56], and multiple comorbid conditions [32].

## 3. Asthma-COPD Overlap Syndrome

The *asthma-COPD overlap syndrome* is not clearly defined. It is a syndrome in which older adults [7] with a significant smoking history have asthmatic features to their chronic obstructive airway disease. Individuals may have a history of childhood asthma or asthma as young adults. There are many cases of pathologic and functional overlap between asthma and COPD [11], yet authorities debate whether this overlap syndrome represents the coexistence of two common airway diseases or whether there are common underlying pathogenic mechanisms leading to this common phenotype [15]. Overlap syndrome appears to share many of the same disease risk factors as that of asthma and COPD (Figure 6). This is a major challenge to our understanding of pathogenesis; however, this observation can guide future basic studies and the development of novel therapies.

The exact definition of this syndrome is evolving. For example, Gibson and Simpson defined overlap syndrome as asthma and COPD, that is, “symptoms of increased variability of airflow and incompletely reversible airflow obstruction,” [34] but this is rather limited when based solely on FEV_1_ and bronchodilator response. For example, patients with COPD can demonstrate a variable and significant degree of reversibility of airflow obstruction following bronchodilator challenge. Nearly 66% of COPD patients in the UPLIFT trial improved their FEV_1_ by more than 15% after receiving 80 *μ*g ipratropium and 400 *μ*g of salbutamol, yet they were not considered to have overlap syndrome or concomitant asthma [57]. Thus, a clear definition remains elusive regarding overlap syndrome since there is no consensus in the literature. Moreover, the presence of exercise intolerance and static or dynamic hyperinflation and indices of pulmonary emphysema in the aging patient may be important factors to include when considering overlap syndrome—a complex question needing further research. 

Few studies have specifically investigated the asthma-COPD overlap syndrome [3, 7]. Epidemiologic studies report an estimated prevalence of 20% [7, 8]. Patients with coexisting asthma and COPD present similarly to pure asthma or COPD, manifesting signs and symptoms of obstructive lung physiology. However, patients with overlap syndrome have worse lung function, more respiratory symptoms, and a lower health-related quality of life than either disease alone [58, 59]. They also consume more medical resources compared to asthma or COPD alone, as much as 2 to 6 times higher [3]. From a cost perspective alone (besides the importance of proper diagnosis and treatment), there is ample reason to increase our research efforts in this area.

Genetic linkage studies and genome-wide association (GWA) studies have been of limited value in characterizing a link between asthma and COPD. According to Postma et al., the paucity of GWA studies, assessing overlap syndrome and the focus on clearly defined outcomes which do not include overlap, hampers the current insight genetic studies provide [60]. In keeping with the Dutch Hypothesis, perhaps studying the genes related to airway development (e.g., Wnt pathway) and how these genes are altered in subjects with asthma [61] and COPD [62] may provide clues to linkage. One notable feature in many genetic and clinical studies is that precise definitions of asthma-COPD overlap simply do not exist. In order to understand the exact nature (genetic or otherwise) of the asthma-COPD overlap syndrome, we must be able to better define the entity.

Cigarette smoking interacts with the inflammation and remodeling that occur in asthma and COPD [11]. The diseases are *most different* when nonsmoking asthmatics with AHR and smokers with COPD but no AHR are compared. Smoking influences the pattern of inflammation and steroid responsiveness [63, 64]. Asthmatics who smoke have more neutrophils in their airways, rather than eosinophils, resembling COPD [63]. Smoking promotes neutrophilic inflammation in both asthma and COPD which results in increased steroid resistance [65, 66]. Disease severity increases as the patterns of inflammation become more similar and steroid resistance increases. Similarly, mucosal eosinophils increase in acute exacerbations of mild COPD, a feature normally seen in asthma [11]. This similarity in inflammatory responses may be one *pathophysiologic link* to the clinical phenotype of asthma-COPD overlap. Despite published definitions of asthma and COPD by international respiratory societies such as ATS/ERS [4] and global initiatives such as GINA [67] and GOLD [2], there remains considerable clinical and pathologic overlap between these two disorders which defies such limited definitions [35].

Overlap syndrome is more prevalent in the aging population. In general, lung function tends to deteriorate naturally with increased age. Elderly patients with asthma display more features of fixed obstruction than their younger counterparts, and they tend to have more severe symptoms [42, 68]. Their asthma may manifest as chronic persistent airflow obstruction mimicking COPD [69]. AHR also increases with age where it is three times higher in the elderly compared to nonelderly adults [42]. Age is a very important variable when assessing obstructive lung diseases given the known changes in lung function that occur with increased age [70] and the possible role of aging genes especially in COPD [71]. Because of this age effect, appropriate comparisons between asthma and COPD should be made in patient cohorts with the same or similar age, inhaled exposures, and disease severity. Indeed, increasing age may be a powerful factor “blurring the line” that separates asthma from COPD, thus, contributing to the manifestation of overlap syndrome.

## 4. A Diagnostic Dilemma: Our Experience

In our academic pulmonary clinics at the University of California, Davis Medical Center (UCDMC), we have observed a significant proportion of patients who present with an overlap of asthma and COPD. *We defined the overlap syndrome as one of two clinical phenotypes:* (1) allergic disease consistent with asthma, that is, variable airflow obstruction or AHR that is incompletely reversible (with or without emphysema or reduced carbon monoxide diffusion capacity (DL_CO_)) or (2) COPD with emphysema accompanied by reversible or partially reversible airflow obstruction (with or without an allergic syndrome or reduced DL_CO_). Patients must have demonstrated either one of these to be diagnosed with “overlap syndrome.” Reversible airflow obstruction is traditionally defined as an increase in FEV_1_ or FVC by ≥200 mL and 12% postbronchodilator challenge, and AHR is defined by a positive methacholine challenge test. For those with clinical findings consistent with an asthma or COPD diagnosis, we followed internationally agreed-upon definitions. Asthma was defined by the GINA executive summary criteria [67], as a clinical syndrome with “variable, airflow obstruction within the lung that is often reversible either spontaneously or with treatment.” And COPD was defined by the joint ATS/ERS task force [4], as “a preventable and treatable disease state characterized by airflow limitation that is not fully reversible.” We used the ATS/ERS recommended lower limit of the normal range for FEV1/FVC ratio (based on the 5th percentile corrected for age, gender, height, and ethnicity) to detect abnormal expiratory airflow obstruction in order to avoid overdiagnosis of COPD.

**Figure 1:**
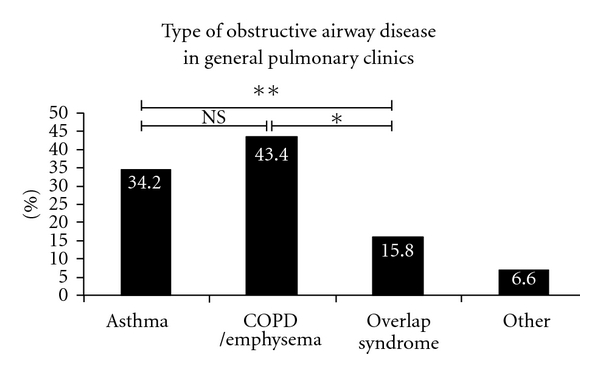
The prevalence of obstructive airway diseases in a small cohort of general pulmonary clinic patients at the UC Davis Medical Center. The category of “Other” represents a combination of bronchitis, bronchiectasis, bronchiolitis, and/or cystic fibrosis cases. The numbers within each bar above represent the percentage for that group (*P = 0.0003 and **P = 0.014 by Fisher's exact test). For definitions of the different diagnostic groups, please see the text.

**Figure 2:**
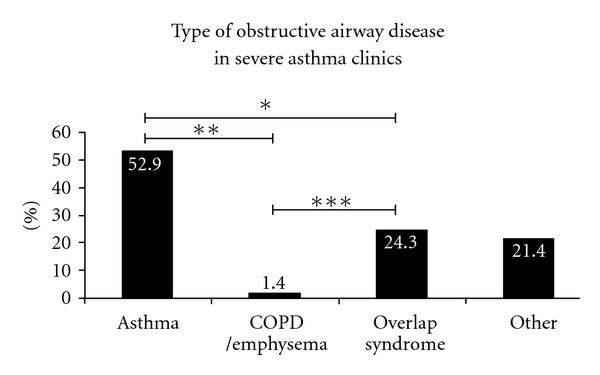
The prevalence of obstructive airway diseases in a small cohort of patients from our the UC Davis Asthma Network (UCAN) severe asthma clinic at the UC Davis Medical Center. The category of “Other” represents a combination of bronchitis, bronchiectasis, and/or bronchiolitis. The numbers within each bar above represent the percentage for that group (*P = 0.0009, **P < 0.0001, and ***P < 0.0001 by Fisher's exact test). For definitions of the different diagnostic groups, please see the text.

**Figure 3:**
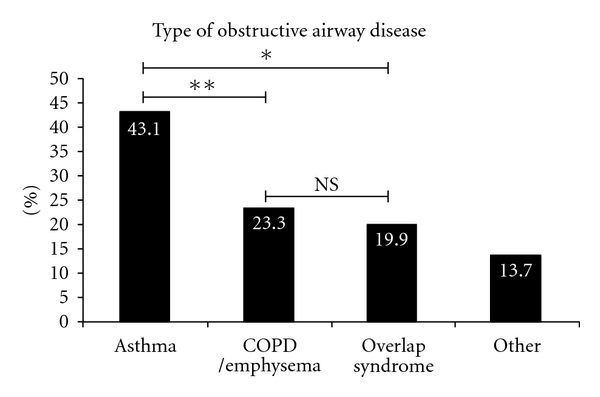
The prevalence of obstructive airway diseases in our combined cohort of general pulmonary and UCAN severe asthma clinic patients (*N* = 146) at the UC Davis Medical Center. The category of “Other” represents a combination of bronchitis, bronchiectasis, bronchiolitis, and/or cystic fibrosis cases. The numbers within each bar above represent the percentage for that group (*P < 0.0001 and **P = 0.0005 by Fisher's exact test). For definitions of the different diagnostic groups, please see the text.

The following data do not reflect the entire patient population in our academic general pulmonary or asthma referral clinics but a smaller yet representative subset selected for analysis. In the general pulmonary clinic cohort (Figure 1), the prevalence of overlap syndrome is half that of asthma (15.8% versus 34.2%, *P* = 0.014) and slightly over one-third that of COPD/emphysema (15.8% versus 43.4%, *P* = 0.0003), representing a sizable number of patients. Similarly in our severe asthma clinics (the UC Davis Asthma Network (UCAN) Clinics, Figure 2), the prevalence of overlap syndrome is approximately half that of asthma (24.3% versus 52.9%, *P* = 0.0009), again representing a significant proportion of patients with mixed disease or “overlap.” Although asthma is the most prevalent type of airway disease (43.1%), when these data are assessed in aggregate (Figure 3), the number of subjects with overlap syndrome approaches that of COPD/emphysema (19.9% versus 23.3%, *P* = NS). This simple observation underscores the commonplace diagnosis of overlap syndrome in subspecialist pulmonary clinics at UCDMC. 

**Figure 4:**
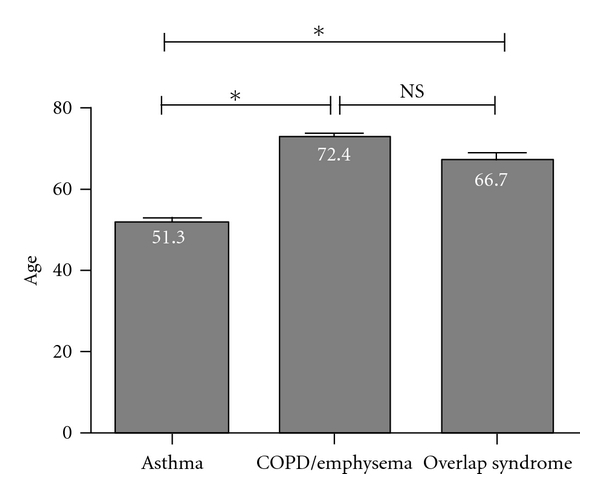
The relative ages of the combined cohort of general pulmonary and UCAN severe asthma clinics at the UC Davis Medical Center. The numbers in each bar represent the mean age per group (*P < 0.0001 by the Kruskal-Wallis test). For definitions of the different diagnostic groups, please see the text.

**Figure 5:**
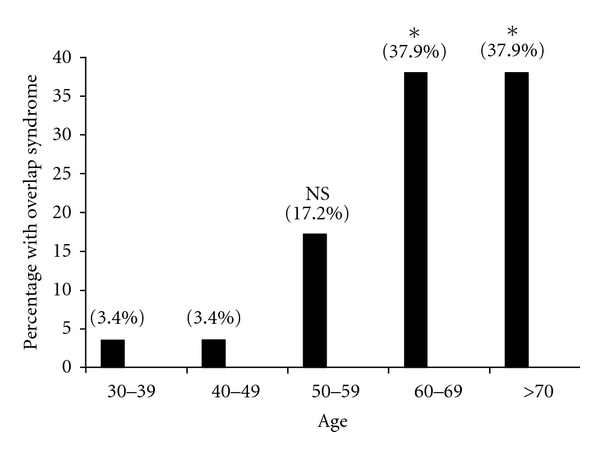
The age distribution of the *N* = 29 subjects diagnosed with overlap syndrome in the combined general pulmonary and UCAN severe asthma clinics at the UC Davis Medical Center. The values above each bar represent the percentage of patients within the defined age group (*P = 0.0024 by Fisher's exact test).

Patients with asthma tend to be younger (mean age 51.3 years old), those with COPD or emphysema tend to be older by two decades (mean age 72.4 years old), and those with overlap syndrome are in between (mean age 66.7 years old). The overlap group, however, was not significantly younger than the COPD/emphysema group (66.7 versus 72.4 years old, *P* = NS; Figure 4). This is consistent with published observations that patients with overlap features tend to be older (i.e. ≥ age 60), at least as compared to patients with asthma. As the population ages, the prevalence of overlap syndrome increases in each decade; the highest prevalence being in those >60 years of age in our cohort (Figure 5). Our observation that overlap syndrome prevalence increases with age mirrors the data reported in much larger cohorts of obstructive lung disease [7, 34]. These data underline the importance of this diagnosis in the elderly or aging population and the need to better distinguish amongst the various causes of chronic airflow obstruction, including overlap syndrome. 

## 5. Treatment Approach for the Asthma-COPDOverlap Syndrome

In general, the principles of workup and treatment are similar for asthma, COPD, and the overlap syndrome. Because this syndrome is seen more commonly in older populations, there may be a higher probability of adverse reactions to the various classes of inhaled agents or systemic corticosteroids [72]. Cognitive deficiencies leading to lower medication compliance may be an issue, and general underdiagnosis and undertreatment [42, 73, 74] may occur. Martinez et al. recommend treatment with a ICS/LABA combination, with or without a long-acting anticholinergic agent (i.e., LAMA) [75]. Smoking cessation, oxygen supplementation, pulmonary rehabilitation, and vaccines are all reasonable interventions. At present, there are no randomized clinical trial data to help guide therapeutic interventions in overlap syndrome [7, 53]. 

Based on our clinical experience, we recommend a symptom-targeted approach. For dynamic obstruction and/or hyperinflation, bronchodilators may provide the greatest benefit. Whether LAMAs alone or in combination with LABAs are appropriate in overlap syndrome remains to be elucidated. For patients in whom bronchospasm is described and/or demonstrated, bronchodilators and ICSs are reasonable options. Adjunctive treatments such as leukotriene receptor antagonists, 5-lipoxygenase inhibitors, methylxanthines, or omalizumab deserve further study and should be administered by pulmonary or allergy subspecialists. As the prevalence of overlap syndrome increases with age, targeting nonrespiratory age-related changes which may influence respiratory disease is paramount. This includes targeting nasal obstruction symptoms (due to non-allergic or allergic rhinitis, mucosal dryness, or vasomotor symptoms) with nasal irrigation, nasal steroids, and/or nasal anticholinergics [76, 77]. Additionally, treating co-morbidities such has heart failure or chronic aspiration is important, especially GERD [78] or VCD which can be subclinical. These recommendations are based on our clinical experiences with the overlap population at UCDMC, where optimal treatment strategies still require customization and additional study. 

## 6. Concluding Remarks

**Figure 6:**
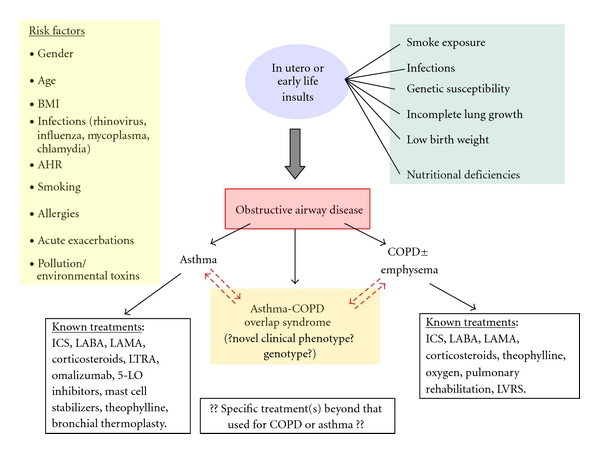
Common risk factors for the development of obstructive airways disease. Although asthma, COPD, and asthma-COPD overlap syndrome**^†^** likely share many of the same risk factors, it is unknown whether the overlap syndrome is a unique genotype and pathophysiologically unique clinical phenotype. The progression from early life insults to pediatric disease and finally chronic obstructive airway disease in adulthood involves complex genetic, epigenetic, and environmental interactions. Because the underlying pathogenic mechanisms that lead to the overlap syndrome have not be elucidated, we have no known disease-specific therapies other than those extrapolated from clinical trials done in asthma- or COPD-only subjects. In this lies an opportunity for further research focused on the overlap syndrome as the “third arm” of the most common obstructive airway diseases. Given that asthma-COPD overlap syndrome prevalence increases with age, knowledge about this syndrome will have great clinical and economic implications for our aging population. **^†^**For definitions of the different diagnostic groups, please see the text. Abbreviations: inhaled corticosteroid (ICS), long-acting beta-agonist (LABA), long-acting muscarinic antagonist (LAMA), leukotriene receptor antagonist (LTRA), 5-lipoxygenase (5-LO), body mass index (BMI), airway hyperreactivity (AHR), and lung volume reduction surgery (LVRS).

Enough evidence exists to suggest that the current definitions of asthma and COPD fail to capture the diverse range of disease phenotypes. COPD, for example, is likely a heterogeneous group of diseases with different genetic backgrounds and anatomic sites of pathology [79], and asthma is no exception [38]. Whether the asthma-COPD overlap syndrome is a separate entity or a hybrid point within a spectrum of related diseases remains to be determined. One certainty is that the overlap syndrome is clinically relevant with a 20% prevalence in populations with airway diseases (similar to 15.8% in our general pulmonary clinics, see Figure 1). One proposal for improved understanding is to assess similar-age patients with overlap features and well-characterized small airway involvement. This would allow for a more homogenous group with similar sites of pathologic airway involvement that can be biopsied and correlated functionally and clinically. Improved clinical data from this subpopulation of obstructive airways disease may reveal previously underappreciated pathophysiologic signatures and create an opportunity for new and targeted therapies (Figure 6). 

Earlier screening of the overlap syndrome is important in current or former smokers in their 5th decade of life who have partially reversible airways obstruction and progressive exercise intolerance and variable (or no response) to guideline recommended asthma treatments. This approach can provide more time to conduct pharmacologic and non-pharmacologic interventions before patients become elderly or develop severe disease. 

Future investigations should include genetic studies, assessment of aging genes expression, inflammatory profiles, exhaled nitric oxide and other biomarkers, and airway biopsies to assess immune cell profiles and airway remodeling. These data should then be correlated to historical (including environmental exposure and other risk factors), clinical, demographic, and physiological parameters. This will help determine whether overlap syndrome is a unique phenotype of obstructive lung disease. Currently, the treatment of overlap syndrome is extrapolated from guidelines for asthma or COPD. It is unknown whether these common inhaled drugs (ICS, LABAs, LAMAs), oral corticosteroids, leukotriene receptor antagonists, and so forth are effective or appropriate to treat this syndrome. Therefore, future basic science studies and multicenter clinical trials should investigate disease mechanisms while developing novel therapies. By studying only those with “pure” asthma or COPD (in our clinical trials) while excluding those with overlapping features, we ignore a considerable proportion of patients with obstructive lung disease. With the aging of our population and the high prevalence and cost of overlap syndrome, clinical trials and further research are needed to investigate this unique population of lung disease.

## Abbreviations

COPD: Chronic obstructive pulmonary disease
NS: Not significant
PFT: Pulmonary function test
DL_CO_: Carbon monoxide diffusion capacity
UCAN: University of California, Davis Asthma Network Clinic
UCDMC: University of California, Davis Medical Center
AHR: Airway hyperreactivity
FEV_1_: Forced expiratory volume in the first second
FVC: Forced vital capacity
ICS: Inhaled corticosteroid
LABA: Long-acting beta-agonist
LAMA: Long-acting muscarinic antagonist
TGF*β*: Transforming growth factor-beta
MMP: Matrix metalloproteinase
T_h_1, T_h_2, T_h_17: Helper T-cell 1, 2, or 17
GWA: Genome-wide association
ATS: American Thoracic Society
ERS: European Respiratory Society
GINA: Global Initiative for Asthma
GOLD: Global Initiative for Chronic Obstructive Lung Disease
GERD: Gastroesophageal reflux disease
VCD: Vocal cord dysfunction.
